# Temperature Conditions in Artificial Sea Turtle Nests: Toward Optimized Hatchery Management

**DOI:** 10.1002/ece3.71750

**Published:** 2025-07-09

**Authors:** Jennifer A. Carbonell Ellgutter, Ingrid Maria Bik, Hans Renssen, Frank Rosell, Lucy A. Hawkes, Stefanie Reinhardt

**Affiliations:** ^1^ Department of Natural Sciences and Environmental Health, Faculty of Technology, Natural Sciences and Maritime Sciences University of South‐Eastern Norway Bø Telemark Norway; ^2^ Section of Grid Licensing The Norwegian Water Resources and Energy Directorate Oslo Norway; ^3^ Marine Turtle Research Group, Centre for Ecology and Conservation, School of Biosciences University of Exeter, Cornwall Campus Cornwall UK

**Keywords:** climate change, ex situ conservation, marine turtles, Olive Ridley, sex ratio, temperature‐dependent sex determination

## Abstract

Climate change poses a significant threat to species with temperature‐dependent sex determination, such as sea turtles. Their conservation often involves relocating nests to hatcheries, which is also crucial on the Pacific coast of Guatemala, where virtually no hatchlings emerge from natural nests. Populations there rely heavily on hatcheries, yet nest temperature monitoring in relation to environmental and management factors is rarely conducted. Research is needed to improve artificial nest management and hatchery design. This study investigated how distance to the hatchery wall, number of eggs, position in the nest, development period, season, and weather conditions influenced temperature variation in Olive Ridley Turtle ( *Lepidochelys olivacea* ) nests. We generally found nest temperatures within viable ranges and near the pivotal temperature for Olive Ridleys. The pivotal temperature of Olive Ridley was exceeded 6%–21% of the time during the thermosensitive period of all nests (starting days 9–15 and ending days 33–37 of incubation), and the upper thermal tolerance limit was rarely reached. However, nests closer to concrete walls were up to 1°C warmer than those farther away, and 30–40 more eggs per nest raised average temperatures by 0.7°C. These findings suggest that distance to hatchery walls and egg numbers per nest can be tools to manipulate nest temperatures and sex ratios. The sex ratios in this study were slightly female‐biased. However, optimal sex ratios remain poorly understood, and reliance on ex situ incubation may reduce population adaptability to environmental changes. Ex situ nest conditions in our study displayed lower temperatures than potential in situ conditions, which exceeded the lethal threshold in 86% (*z*‐test, *p* < 0.001) of the measurements. Our study emphasizes the need for careful hatchery management to safeguard sea turtles against the effects of climate change but also to avoid the consequences of overcompensation due to mismanagement.

## Graphical abstract

This study examined how environmental and management factors, such as wall distance and egg numbers, influence nest temperatures of Olive Ridley sea turtles in a hatchery on the Pacific coast of Guatemala.

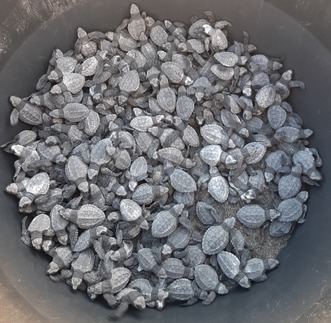

## RESUMEN

El cambio climático representa una amenaza importante para las especies con determinación sexual dependiente de la temperatura (TSD), como las tortugas marinas. Su conservación a menudo implica la reubicación de nidos en viveros, lo cual es crucial en la costa del Pacífico de Guatemala, donde prácticamente no emergen crías de nidos naturales. Las poblaciones en esta región dependen en gran medida de los viveros, aunque el monitoreo de la temperatura de los nidos en relación con factores ambientales y de manejo rara vez se realiza. Se necesita investigación para mejorar el manejo de nidos artificiales y el diseño de los viveros. Este estudio investigó cómo la distancia a la pared del vivero, el número de huevos, la posición en el nido, el periodo de desarrollo, la temporada y las condiciones climáticas influyen en la variación de temperatura en los nidos de tortuga golfina (*Lepidochelys olivacea*). En general, encontramos temperaturas dentro de los rangos viables y cercanas a la temperatura pivotal para la especie. La temperatura pivotal fue superada entre el 6% y el 21% del tiempo durante el periodo termosensible de todos los nidos (entre los días 9–15 y 33–37 de incubación), y el límite superior de tolerancia térmica raramente fue alcanzado. Sin embargo, los nidos más cercanos a las paredes de concreto fueron hasta 1°C más cálidos que los más alejados, y la presencia de 30–40 huevos adicionales por nido elevaron la temperatura promedio en 0.7°C. Estos hallazgos sugieren que la distancia a las paredes del vivero y el número de huevos por nido pueden utilizarse como herramientas para manipular la temperatura de los nidos y las proporciones de sexos. Las proporciones de sexos en este estudio mostraron una leve tendencia hacia hembras. Sin embargo, las proporciones óptimas de sexos siguen siendo poco conocidas, y depender exclusivamente de la incubación *ex situ* podría reducir la adaptabilidad poblacional frente a los cambios ambientales. Las condiciones de los nidos *ex situ* en nuestro estudio presentaron temperaturas más bajas que las condiciones potenciales *in situ*, las cuales superaron el umbral letal en el 86% de las mediciones (*z*‐test, *p* < 0.001). Nuestro estudio enfatiza la necesidad de una gestión cuidadosa de los viveros para proteger a las tortugas marinas contra los efectos del cambio climático, pero también para evitar las consecuencias de una sobrecompensación por mala gestión.

## 1 Introduction

Species with temperature‐dependent sex determination (TSD) are particularly vulnerable to rising temperatures associated with climate change, as their embryo development and sex are dependent on incubation temperatures (Santidrián Tomillo and Spotila 2020). As temperature rises, primary sex ratios can become increasingly skewed, which may change long‐term population viability (Heppell et al. 2022). Increases in temperature not only affect the sex ratio of species with TSD but can also negatively influence the survival of their offspring (Reneker and Kamel 2016; Mainwaring et al. 2017). Additionally, ectothermic species with TSD are especially susceptible to climate change, as environmental temperature variations determine their movement, growth, and reproduction (Deutsch et al. 2008; Angilletta Jr. and Angilletta 2009; Böhm et al. 2016). Furthermore, if the species are egg‐laying, extreme weather events such as extreme heat events, flooding, and droughts are likely to further impact reproduction (Reneker and Kamel 2016; Mainwaring et al. 2017). As the effects of climate change are projected to become more pronounced in the coming decades (IPCC 2018), the threats to ectothermic egg‐laying species with TSD are expected to worsen.

Some species with TSD can likely exhibit plasticity in their nesting behaviors in response to warming (Fuentes et al. 2023). For instance, Chinese alligators (*Alligator sonesis*) and painted turtles ( *Chrysemys picta bellii* ) have shifted their nesting timing, location, and/or depth to counteract climate effects (Zhang et al. 2009; Refsnider and Janzen 2012; Refsnider et al. 2013). However, these adjustments may still be insufficient to fully offset temperature changes, as seen in three‐lined skinks (*Bassiana duperreyi*) (Telemeco et al. 2009) or in some sea turtle populations (Fuentes et al. 2023, 2024; Laloë and Hays 2023). Therefore, in some locations, ex situ conservation has been used to manage endangered species (Mestanza‐Ramón et al. 2020). For example, the critically endangered Chinese alligator has been managed this way for more than 20 years (Zhang et al. 2009), and artificial sea turtle “hatchery” programs are found in many parts of the world (Liles et al. 2019; Shanker et al. 2021).

Sea turtles display TSD during the middle third of development, the thermosensitive period (TSP), with lower temperatures resulting in a higher proportion of male hatchlings and higher temperatures leading to a higher proportion of females (Ackerman 1997; Esteban et al. 2018; Pusapati et al. 2021). Sustained elevated temperatures throughout incubation can also negatively affect hatchling survival and development (Ackerman 1997; Esteban et al. 2018; Pusapati et al. 2021). Rising air temperatures and shifts in precipitation due to climate change may potentially lead to increased sand temperatures on sea turtle nesting beaches, which again may result in higher incubation temperatures. There is growing concern that this could reduce hatching success and hatchling fitness, while also skewing sex ratios increasingly toward females (Cavallo et al. 2015; Türkozan et al. 2023). In some regions, sea turtles nest on dark volcanic sand beaches, such as those in Cape Verde (Laloë et al. 2014), Costa Rica (Espinoza‐Rodríguez et al. 2023), and Guatemala (Ariano‐Sánchez et al. 2023). The dark sand on these beaches has a relatively low albedo compared to light‐colored sand, absorbing more solar radiation and leading to higher sand temperatures. Combined with climate change, these conditions may expose the eggs to prolonged periods of temperatures beyond their thermal tolerance (Laloë et al. 2014; Ariano‐Sánchez et al. 2023; Espinoza‐Rodríguez et al. 2023).

Hatcheries are a common ex situ conservation method for sea turtles, protecting the nests from threats such as predation and poaching, and allowing management of sand temperatures to mitigate climate change effects (Patino‐Martinez et al. 2012; Hays et al. 2014). In Guatemala, for example, widespread and unsustainable poaching of turtle eggs led to legal requirements for sea turtle conservation programs to transfer 20% of all eggs laid on beaches into hatcheries to ensure the future of sea turtles in Guatemala (CONAP 2020); thus, hatcheries have been widely used in some locales throughout the world for many decades. However, the long‐term implications of manipulating sex ratios can also be unfavorable, such as reducing the number of nesting females and, consequently, the effective population size (Santidrián Tomillo et al. 2021, 2023). Uniform incubation environments can diminish natural selection pressures, potentially lowering genetic diversity and resilience to environmental changes (Santidrián Tomillo and Spotila 2020; Heppell et al. 2022). Additionally, high nest densities in hatcheries can facilitate the accumulation of pathogens, which can adversely affect hatching success (Mortimer 1999; Hoh et al. 2020). Therefore, while hatcheries play a role in conservation, careful management is essential to avoid these potential negative impacts (Lockley and Eizaguirre 2021; Tanabe et al. 2021).

Despite these possible negative consequences, sea turtle hatcheries are often considered a useful ex situ conservation tool (Martins et al. 2021). They do not only mitigate climate change effects but are also applied to prevent sea turtle nests from predation, egg poaching, and other human activities or environmental fluctuations (Lutcavage 1997; Martins et al. 2021; Pusapati et al. 2021). In hatcheries, nests are usually protected from these threats, and they allow for thorough monitoring of incubation conditions. Nests in hatcheries are, to a varying degree, shielded from the outer environmental impacts, but many external factors, such as weather conditions (precipitation, air temperature, radiation) do still have an impact on the nests inside the hatcheries (Fuentes and Porter 2013; Staines et al. 2020; Van De Merwe et al. 2006). Mitigation actions to address climate change are artificial shading (Wood et al. 2014), deeper nest depth (Van De Merwe et al. 2006), and irrigation (Hill et al. 2015), leading to lower temperatures in the nests inside the hatcheries compared to in situ nests. However, the optimal sex ratio for long‐term population viability remains unknown. While a moderate female bias may support population growth, excessively skewed sex ratios could reduce genetic diversity and reproductive resilience (Wibbels et al. 1998; Hays et al. 2017). Therefore, further research on conditions within hatcheries is required to analyze artificial nest management and hatchery design.

In this study, we assessed various factors affecting nest temperature in a sea turtle hatchery on the Pacific coast of Guatemala to understand the artificial incubation regime and its relation to climate predictions and management actions. We hypothesize that the position of the nests in the hatcheries influences nest temperatures, predicting higher temperatures in nests closer to the hatchery walls compared to nests further away from the walls. Additionally, we tested the impact of egg numbers per nest, time of development, position within the nests, and external environmental factors on nest temperatures in the hatcheries. We also compared the ex situ sand temperature to the in situ sand temperature on the beach to better understand the actual conditions in and outside the hatchery.

## 2 Material and Methods

### 2.1 Study Area and Sea Turtle Hatchery

**FIGURE 1:**
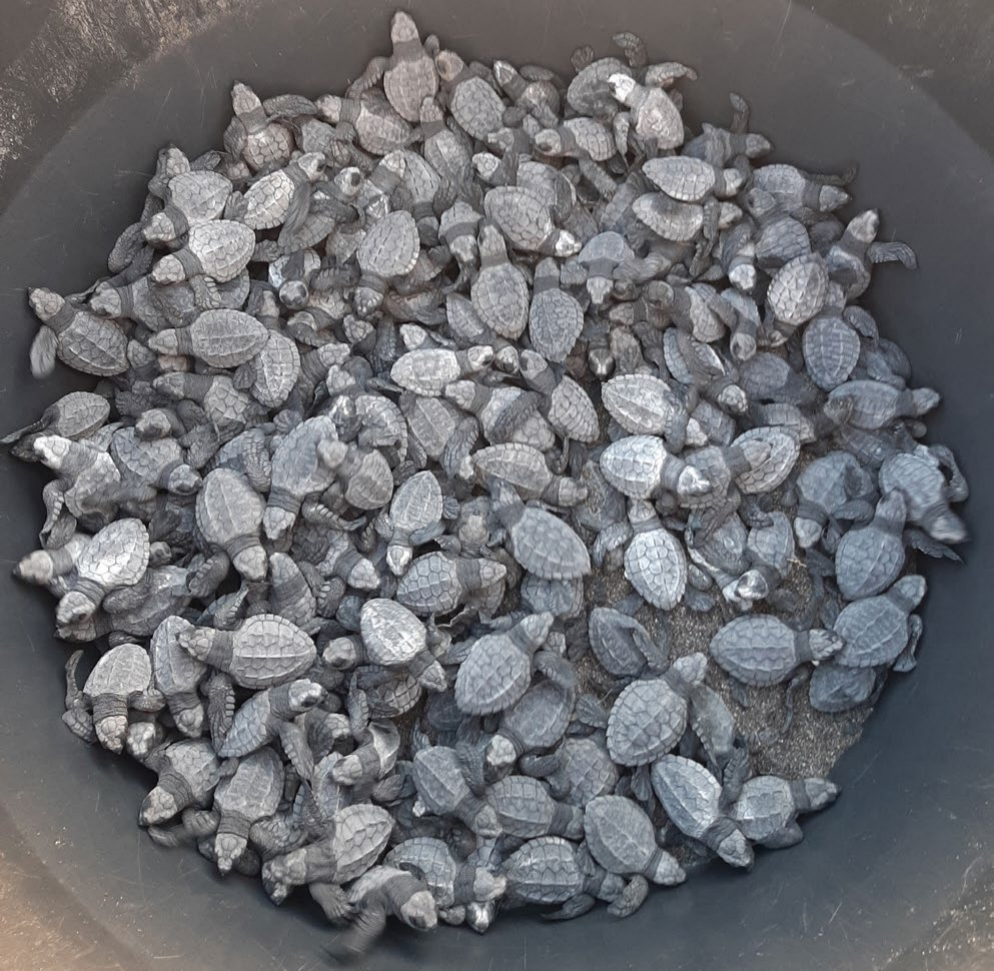
Olive Ridley sea turtle (*Lepidochelys olivácea*) hatchlings from the hatchery El Banco, Guatemala, placed in a black bucket and awaiting release into the ocean as part of the conservation management process in 2022.

On the Pacific coast of Guatemala, sea turtle eggs are rarely incubated in situ because local egg collectors harvest most of the eggs from the nesting beaches to sell them on food markets or to deliver them to hatcheries. The practice of relocating eggs to hatcheries began in 1971 when locals noticed a significant decline in the number of nesting turtles (MARN and PNUD 2018). By 2017, the sea turtle population was still decreasing, prompting the creation of a Guatemalan law that mandates 20% of harvested eggs to be delivered to hatcheries. Some hatcheries also buy the remaining eggs to prevent them from being sold on markets. Today, there are around 34 active sea turtle hatcheries on the Pacific coast of Guatemala, and nearly all eggs delivered to the hatcheries are from the Olive Ridley Turtle (*Lepidochelys olivaceae*; Figure 1) (CONAP 2018, 2020; Ariano‐Sánchez et al. 2020; Morales Mérida et al. 2021).

Olive Ridley Turtles have a pivotal temperature (incubation temperature that produces a 1:1 of each sex) that typically ranges from 29.5°C to 31.0°C (with an average of 30.3°C), varying across different regions and populations, with a thermal tolerance of the embryos spanning from 24°C to 36°C (Casthelogue et al. 2018; Liles et al. 2019; Tello‐Sahagún et al. 2023). In experimental studies, the TSP of Olive Ridley Turtles has been shown to be around the middle third of incubation, which is used as an approximation of the middle third of development (Merchant‐Larios et al. 1997). However, embryo development is dependent on incubation temperatures (Booth 2017; Rutledge et al. 2024). Therefore, the TSP can vary from nest to nest. On the Pacific coast of Guatemala, Olive Ridleys nest all along the coast, with higher nesting density in the southeast, with most hatcheries located in this area (Muccio 2019).

**FIGURE 2:**
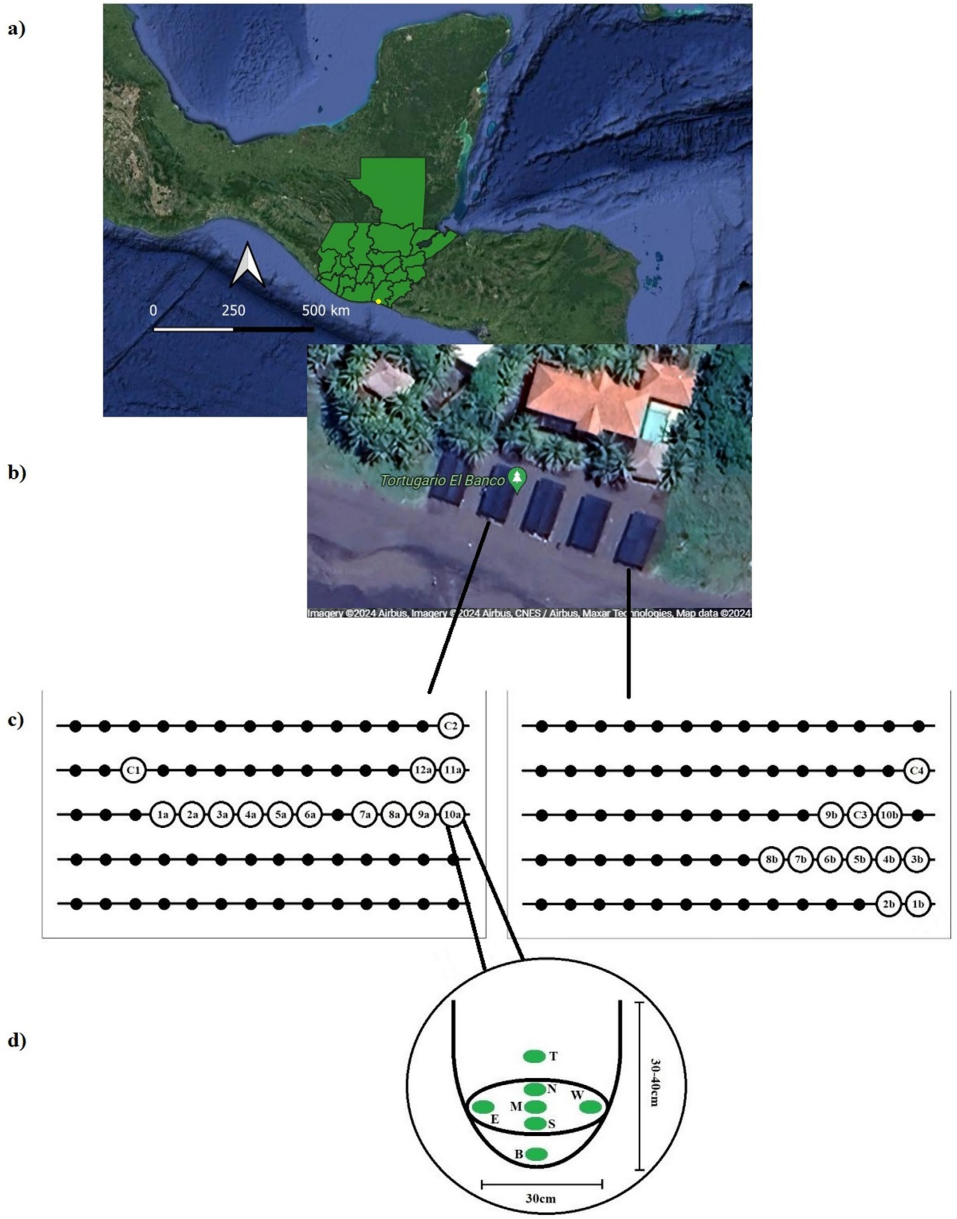
(a) Map of Guatemala, the yellow dot marks the location of the hatchery at El Banco beach (Tortugario El Banco) on the Pacific coast of Guatemala (OCCHA FISS 2019). (b) The picture shows the hatchery with the five buildings where nests are buried (Maps 2024). (c) The black dots represent the nests in the hatchery. The nests used in this study are the labeled circles. C1–C4 are control nests (temperature measurements from empty nests). (d) Nest dimensions and the placement of the data loggers (green circles) in B—bottom, S—south, E—east, W—west, N—north, M—middle, and T—top of each nest.

**FIGURE 3:**
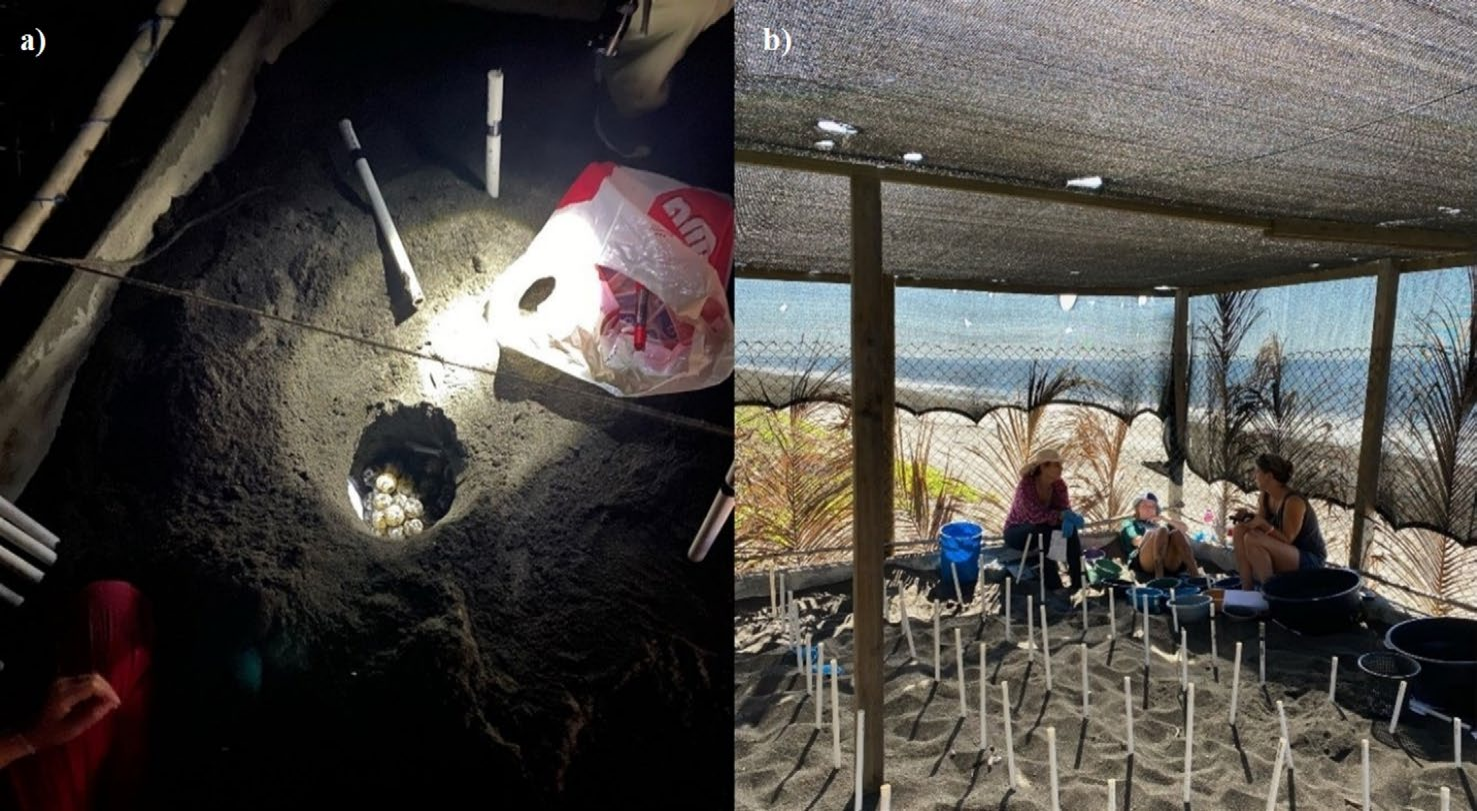
(a) A nest inside the hatchery before closing it inside El Banco hatchery. (b) Inside of the hatchery where each nest is marked by the white PVC plastic pipes.

Our study was conducted in El Banco, a sea turtle hatchery situated at the edge of a sandy beach on the southeast Pacific coast of Guatemala (13.90339° N, −90.52531° W) (Figure 2). In Guatemala, this hatchery has been receiving the highest number of eggs in recent years (Muccio 2019). El Banco consists of five buildings, each spaced five meters apart, with their respective perimeters built on a foundation of concrete blocks measuring 15 cm in width, approximately 60 cm in height above the surface, and buried 40 cm beneath the sand. The hatchery is surrounded by wire mesh walls on top of the concrete perimeter, with wooden support posts (supplemented with palm leaves during the dry season, November 10th onwards) and topped with a 50%–80% black saran shade cloth (Figure 3) (Muccio 2019). During the dry season, additional shading was provided by placing palm leaves along the sides of the hatchery structures. Freshwater irrigation was also applied using garden sprinklers positioned outside the enclosures; however, no records were kept regarding the frequency of irrigation. The hatchery contains black volcanic sand sourced from the beach, which is refreshed every second year. Approximately 50–110 eggs (Lamar 1986; López‐Castro et al. 2004; Plotkin 1994) are buried per hole of 30–40 cm deep and 20–30 cm wide, attempting to mirror natural Olive Ridley Turtle nests (Valverde et al. 2010; Dornfeld et al. 2015). Each nest is marked with PVC plastic pipes (Figure 3). The distance from the nests closest to the wall is 50 cm, and the distance between nests is 40 cm. The typical incubation period ranges from 45 to 65 days (Hill et al. 2015), with the hatchery records showing it is usually closer to 45 (“Estación Biológica El Banco” 2023). This period can vary depending on location and nest temperature, as elevated nest temperatures often shorten it (Booth 2017; Pusapati et al. 2021). However, in the hatchery, hatchlings do not emerge by themselves but are dug out by the hatchery staff on day 45. If the nest is not ready, the eggs are put back into the nest and again covered by sand. Thus, very few hatchlings emerge from the nest independently. Nesting turtles can be found all year round, but most eggs are delivered to the hatchery between July and December (Muccio 1998; CONAP 2020), peaking between August and October (Handy 2005). The local climate alternates between a rainy season from May to October and a dry season from November to April (Holder 2004; Ruano and Milan 2014; Anchukaitis et al. 2015). Monthly air temperatures average between 23.9°C and 30°C, with an annual mean of 27°C and a peak of 30°C in April (Muccio et al. 2010; Ariano‐Sánchez et al. 2023).

### 2.2 Data Collection

Incubation temperature was recorded in 22 nests over two seasons in 2021 (September to October for the rainy season, and November to December for the dry season). Log‐Tag TRIX‐8 temperature dataloggers (range −40°C to +85°C, accuracy ±0.5°C for −20°C to +40°C) were placed at seven positions (the bottom, middle, top, and the sides of the nest named south, east, west, north) within each nest (mean number of eggs per nest: 80 ± 18 SD in the rainy season, and 81.6 ± 15 in the dry season), located at different positions in the hatcheries (Figure 2). The data loggers were programmed to record temperature hourly throughout the entire incubation period. Sand temperature was also measured in two control nests (at 30–40 cm depth), i.e., nests without eggs, throughout the season. After 45 days of incubation, loggers were removed from the nests and downloaded. All fully developed hatchlings from each nest (*n* = 1210) were exhumed and measured using a digital caliper to the nearest 0.1 mm. Any dead hatchlings were collected, and 40 from the rainy season were randomly chosen and sexed via examination of the gonads (following Mrosovsky and Benabib 1990). Hourly weather data (precipitation, air temperature, and solar radiation) were obtained from the Instituto Privado de Cambio Climático (ICC) network, from an automatic weather station in La Candelaria, located at the beach, 1 km southwest of El Banco hatchery.

### 2.3 TSP and Sex‐Ratio

The hourly temperature data was downloaded through LogTag Analyzer software. Using the embryogrowth package (Girondot, Guillon, and Godfrey 2018), we modeled embryonic development over time, which allowed us to estimate the TSP for each nest based on proportional development, defined from stage 0.21 to 0.71, with stage 1 representing a fully developed hatchling (Girondot, Monsinjon, and Guillon 2018). We also divided the incubation period into three development periods: early third, middle third (TSP), and late third. Based on the mean temperature during the TSP, we estimated the proportional sex ratio for each nest using a logistic model with a pivotal temperature of 30.3°C and a slope of −0.2 (Wibbels et al. 1998; Girondot, Monsinjon, and Guillon 2018).

### 2.4 Factors Influencing Nest Temperatures

The effect of eight different factors on nest temperature was analyzed using generalized additive mixed models (GAMMs), which allow a non‐linear relationship with the response variable changing throughout the days and months (He et al. 2006; Zuur 2012). Candidate models were created to assess the effects of the following factors: location in the hatchery in relation to seven increasing distances from the hatchery wall (50, 90, 130, 170, 210, 250, and 290 cm), number of eggs in each nest, development period (early, middle‐TSP, and late third of development), season (dry and rainy season), position within the nest (bottom, center, top, east, south, west, and north), and local hourly weather represented by air temperature (°C), precipitation (mm), and solar radiation (W/m^2^). The identification number of each logger (loggerID) was used as a random effect to account for spatial dependency. Since the temperature values were recorded hourly over approximately 45 days, there were sufficient unique values to use hour (hour) and Julian day (Jday) as temporal smoothers. However, Jday was correlated (Pearson *r* coefficient > 0.5) (Harrison et al. 2018) with the season and the development period, so we opted to exclude Jday from the models due to the importance of the TSP. Additionally, radiation was highly correlated with air temperature. Since the hatchery is covered with netting to protect the nests from intense radiation, we did not use radiation as a factor in the models. The values of the variance inflation factor were < 3, showing that the factors included in the models were not correlated with each other (Zuur et al. 2010).

We performed a model selection process guided by Akaike's Information Criterion (AIC) (Sutherland et al. 2023). When several potential models shared identical weights, we conducted model averaging to assess the cumulative effect size of the predictor variable (Harrison et al. 2018). Even though other studies have found that temperature distribution within the nest fluctuates during embryo development (Kaska et al. 2000; Luna et al. 2000; Booth and Astill 2001), the variable “position within the nest” was removed from the analyses because it did not contribute to the model (Wagenmakers and Farrell 2004). Finally, the chosen model was validated using residuals and quantile‐quantile plots (Zuur et al. 2010).

Finally, to quantify the effect that hatcheries have on sand temperature, the number of hours that control nests spent above the pivotal temperature and the upper thermal tolerance were compared to data from another study performed at the same time. In that study, we measured hourly sand temperature at 30/50 cm depth at eight locations on a stretch of approximately 15 km of nesting beach between El Banco and El Hawaii (13.86899° N, −90.41932° W). These measurements were also conducted close to and farther away from concrete structures. However, they only represent the measurement of sand temperatures, not nest temperatures. Eggs in the nest are shown to add a metabolic heat of 1.5°C–3.0°C to the temperatures in the nests, depending on different factors such as incubation period and number of eggs (Broderick et al. 2001; Gammon et al. 2020). We chose to add 1.5°C as a conservative approach to the measured sand temperatures as a representative temperature difference for metabolic heat, to allow for comparing in situ measurements in the sand with temperatures in the hatchery nests. All data analyses were performed using R Studio software version 4.3.0 (R Core Team 2022).

## 3 Results

Overall, nest temperatures in the hatchery ranged from 25.8°C to 36.4°C (lowest to highest measurement in the study) and exceeded the pivotal temperature of 30.3°C in 72% of hours during TSP (middle third) (number of nests = 22, *z*‐test, *p* < 0.001). Nests were rarely warmer than the upper thermal tolerance limit of 36°C (0.1% of hours during the incubation period of the studied nests, *z*‐test, *p* < 0.001). Nest temperatures were slightly higher during the rainy season (*n* = 12 nests, 31.1°C ± 1.7) compared to the dry season (*n* = 10 nests, 31.3°C ± 1.6, *t*‐test, *p* = 0.02) (Table 1).

**TABLE 1:** Summary of the mean nest temperatures (°C) with their respective ±SD at Hatchery El Banco, from September to December 2021.

Effect	Level	Mean temperature	Rainy season	Dry season
Third of development	Early	29.8 ± 1.0	29.6 ± 0.9	30.0 ± 1.0
	Middle	30.9 ± 1.0	30.9 ± 1.0	30.9 ± 1.1
	Late	33.4 ± 1.3	33.3 ± 1.4	33.6 ± 1.0
Distance	50 cm	31.7 ± 1.4	31.4 ± 1.3	32.1 ± 1.4
	90 cm	31.6 ± 1.6	31.5 ± 1.7	31.7 ± 1.6
	130 cm	31.1 ± 1.7	31.1 ± 1.7	31.1 ± 1.7
	170 cm	30.7 ± 1.7	30.7 ± 1.7	30.6 ± 1.6
	210 cm	30.5 ± 1.5	30.4 ± 1.6	30.5 ± 1.4
	250 cm	30.9 ± 1.6	31.0 ± 1.7	30.7 ± 1.4
	290 cm	31.2 ± 1.8	31.2 ± 1.8	NA
All nests		31.2 ± 1.7	31.1 ± 1.7	31.3 ± 1.6

*Note:* “Third” represent the development period, divided into three thirds. “Distance” is the gradient from 50 to 290 cm from the wall, were 50 cm is closest to the wall and 290 cm the farthest.

The best‐fitted model for predicting the nest temperature included the variables distance to the hatchery wall, number of eggs, development period, season, air temperature, precipitation, hour as a smoother, and loggerID as a random effect (Table 2). Nest temperature increased with proximity to hatchery walls, the number of eggs, the development period, and precipitation while it decreased with air temperature. Season (rainy or dry) had the least explanatory power on nest temperature (Table 3).

**TABLE 2:** Model selection using Akaike Information Criterion (AIC) and their respective degrees of freedom (Df), and the different variables with their respective sum of weights, where 1 indicates the variable is highly important and 0 the opposite.

Model	Df	AIC	Variable	Sum of weights
Nest Temperature~1	2.0	267,393.9	Air temperature	1
Nest Temperature~s(hour)	8.4	265,686.8		
Nest Temperature~s(hour) + r(loggerid)	71.2	255,172.0	Distance	1
Nest Temperature~s(hour) + distance + r(loggerid)	70.9	255,171.7		
Nest Temperature~s(hour) + distance + number of eggs + third of development + season + r(loggerid)	73.9	171,785.6	Precipitation	1
Nest Temperature~s(hour) + distance + number of eggs + third of development + season + position + r(loggerid)	73.9	171,785.5	Third of development	1
Nest Temperature~s(hour) + distance + number of eggs + third of development + season + position + air temperature + r(loggerid)	75.9	171,663.9	Number of eggs	0.8
Nest Temperature~s(hour) + distance + number of eggs + third of development + season + position + air temperature + precipitation + r(loggerid)	76.8	171,659.4	Season	0.8
Nest Temperature~s(hour) + distance + number of eggs + third of development + season + air temperature + precipitation + r(loggerid)	76.8	171,659.4	Position	0.3
Nest Temperature~s(hour, k = 6) + distance + number of eggs + third of development + season + air temperature + precipitation + r(loggerid)	73.6	171,672.6	s(hour)	1
**Nest Temperature~s(hour, k = 10) + distance + number of eggs + third of development + season + air temperature + precipitation + r(loggerid)**	**76.8**	**171,659.4**	s(loggerid)	1

*Note:* The random effect is “r(loggerid)” and the smoother is “s(hour)”. The bold values in this table is the chosen model

**TABLE 3:** Summary of the Generalized Additive Mixed Model (GAMM) of the nest temperature (°C).

Variables	Estimate	Std. error	*t*	Pr(> |*t*|)
Intercept	29.51	0.330	89.271	< 0.001***
Distance90 cm	−0.312	0.153	−2.040	0.041*
Distance130 cm	−0.742	0.195	−3.799	< 0.001***
Distance170 cm	−0.965	0.154	−6.265	< 0.001***
Distance210 cm	−0.723	0.224	−3.227	< 0.001**
Distance250 cm	−0.683	0.168	−4.055	< 0.001***
Distance290 cm	−0.568	0.211	−2.693	0.007**
Dry season	0.097	0.100	0.970	0.332
Middle third	1.113	0.008	144.882	< 0.001***
Late third	3.607	0.009	391.557	< 0.001***
Number of eggs	0.018	0.004	4.756	< 0.001***
Air temperature	−0.026	0.002	−10.830	< 0.001***
Precipitation	0.020	0.008	2.578	< 0.001**
**Approximate significance of smooth terms**				
	**edf**	**Ref.df**	***F***	***p***
s(hour)	8.083	8.789	626.7	< 0.001***
s(loggerid)	54.754	56.0	223.6	< 0.001***

*Note:* Significant codes: “***” 0.001, “**” 0.01, “*” 0.05, “.” 0.1, “”1. *R*‐sq.(adj) = 0.748. Deviance explained = 74.8%. GCV = 0.691, Scale est. = 0.691, *n* = 69,530.

### 3.1 Nest Position in the Hatchery

**FIGURE 4:**
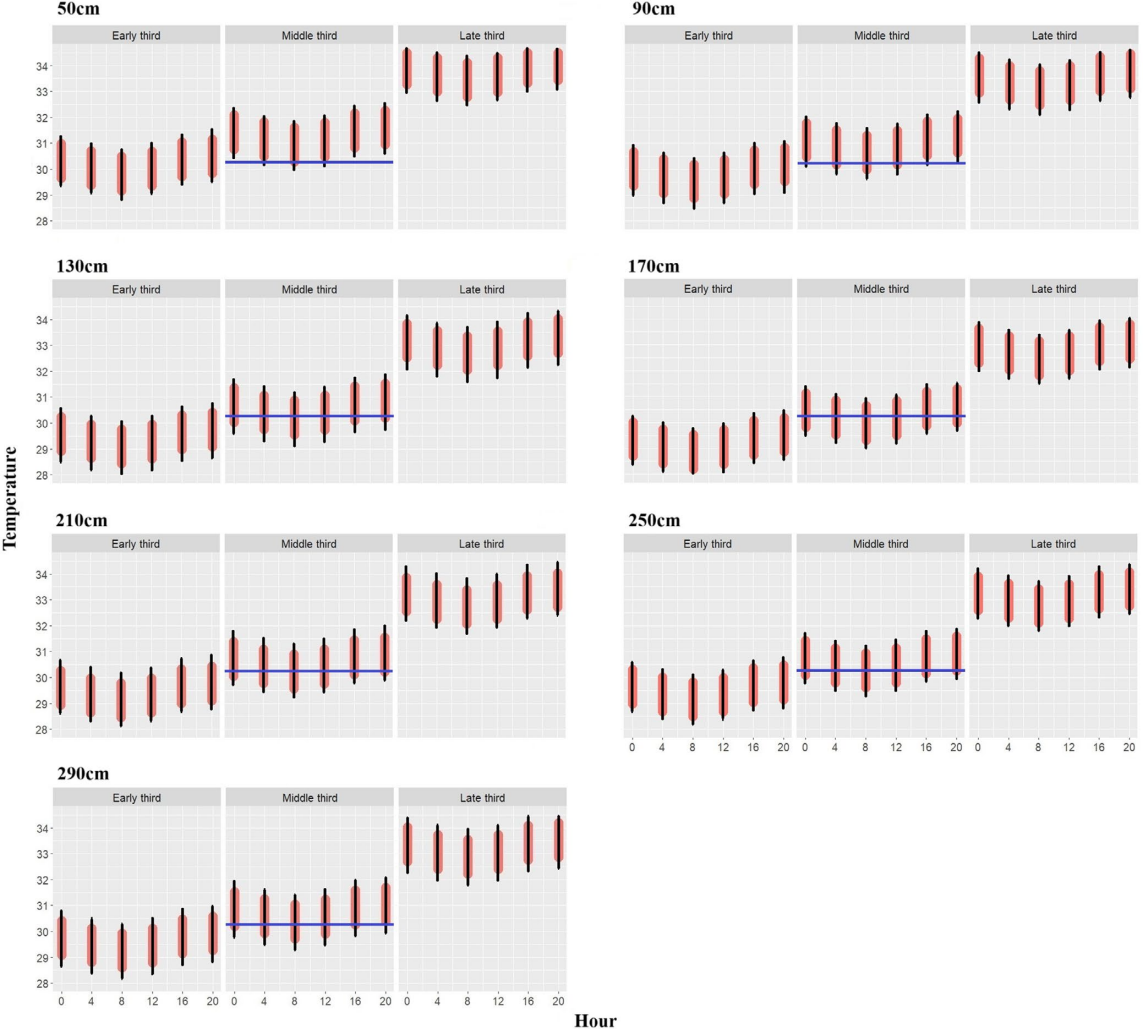
Hourly temperature (°C) variation in the nests by the distance to the wall (50, 90, 130, 170, 210, 250, 290 cm) and the stages of the embryo development separated by three thirds of the development period (early, middle‐TSP and late). The black lines are the 95% confidence intervals. 50 cm represents the nests closest to the wall while 290 cm the farthest away. The blue line in the middle third represents the pivotal temperature for Olive Ridley Turtle (30.3°C).

Nests closer to the concrete hatchery wall (distance: 50 and 90 cm) were above the pivotal temperature most of the hours during incubation time (89% and 80% respectively, ANOVA, *p* < 0.001), while nests located farther from the wall (distance: 250 and 290 cm) were above the pivotal temperature less frequently (58% and 66% respectively, ANOVA, *p* < 0.001) (Figure 4). Further, during the TSP the nests closer to the hatchery walls (50 and 90 cm) spent more hours (19% and 21% respectively, ANOVA, *p* < 0.001) above the pivotal temperature, while nests farther away were above the TSP only 6%–8% (ANOVA, *p* < 0.001) of the time. Compared to nests 50 cm away from the hatchery wall, nests were 0.1°C–0.6°C (ANOVA, *p* < 0.001) cooler when they were 90–130 cm away, and an average of 1.0°C (ANOVA, *p* < 0.001) cooler when they were farther away (Table 1). During the dry season, nest temperature was, on average, 0.5°C (ANOVA, *p* < 0.001) lower in nests that were 290 cm away from the hatchery wall compared to nests that were 50 cm away from the wall (nests that were 290 cm away from the hatchery wall were not measured during the rainy season).

### 3.2 Other Factors Influencing Nest Temperature

**FIGURE 5:**
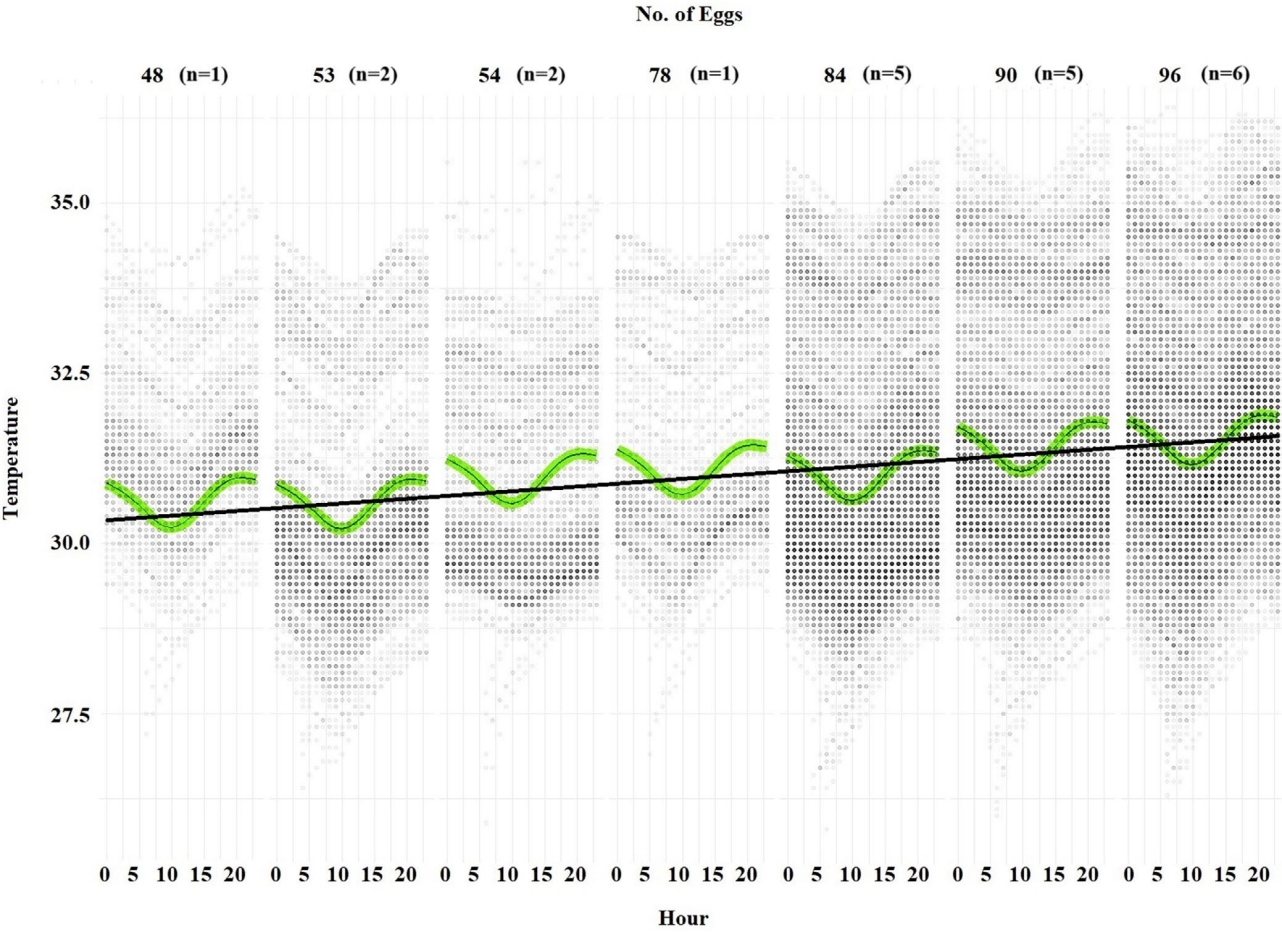
Relationship between nest temperature and number of eggs in the nest (the number in parentheses represents the number of nests for each egg count category). The green lines represent the 95% confidence intervals of the hourly mean temperature by the amount of number of eggs in the nests. The dots represent the raw data.

Daily fluctuations in nest temperature were recorded as seen in Figure 4. Further, we found that nest temperature was also influenced by the number of eggs in the nest, development period, and weather conditions. An increase in the number of eggs was associated with a corresponding rise in nest temperature, e.g., 30–40 more eggs in the nest led to an average increase of 0.7°C (mean 30.7°C ± 1.4 SD vs. 31.4°C ± 1.7, *t*‐test, *p* < 0.001) (Figure 5). Nest temperature also varied with trimesters of the development period. During the late third, nest temperature was 3.6°C warmer than during the early third of the development period (mean 33.4°C ± 1.3 vs. 29.8°C ± 1.0, *t*‐test, *p* < 0.001), likely due to metabolic heat. Air temperature had a negative effect (*p* < 0.001) on the nest temperature, while precipitation had a positive effect (*p* = 0.01) (Table 3).

**FIGURE 6:**
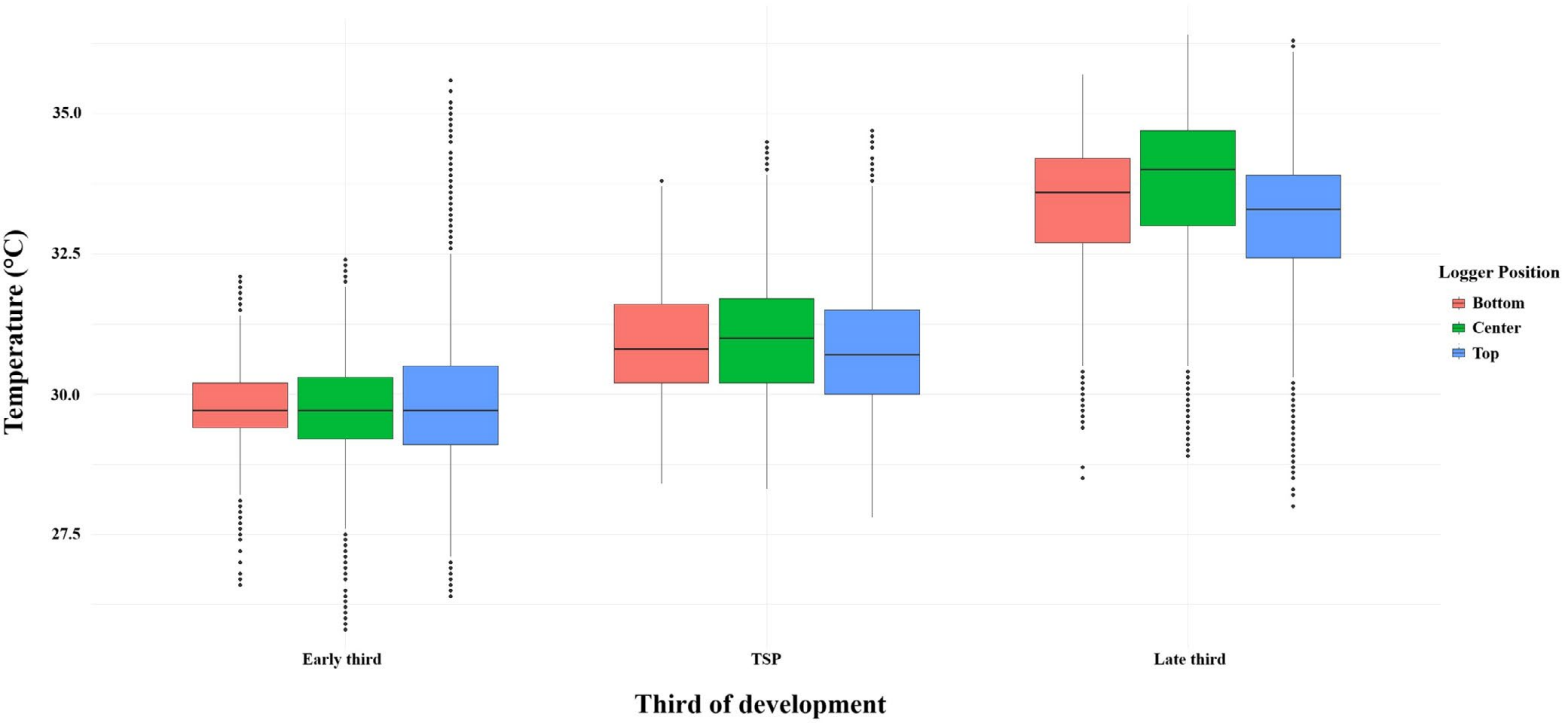
Box plot showing the distribution of nest temperatures recorded at three logger positions within the nest (bottom, center, and top) across the three thirds of development (early, TSP, and late third). Each box plot represents the interquartile range (IQR) of hourly temperature, with the horizontal line marking the median. Whiskers indicate the full data range; the black dots are the outliers.

Within each nest, the temperatures seemed slightly cooler at the center position compared to both the bottom and top positions (Figure 6). During the TSP, however, the center position became warmer than both the bottom and top (+0.1°C and +0.3°C, *t*‐test, *p* < 0.001). This trend intensified in the late third, with the center position being +0.4°C warmer than the bottom and +0.7°C warmer than the top (*t*‐test, *p* < 0.001; Figure 6).

### 3.3 Ex Situ Versus In Situ Sand Temperature

The hatchery sand temperature (measured in control plots without eggs) was, on average, 4.8°C lower than the sand temperature on the nesting beach (mean 30.4°C ± 0.8 vs. 35.2°C ± 0.6, *t*‐test, *p* < 0.001). Sand temperature on the beach was above the pivotal temperature throughout the study period. In contrast, in the hatchery, sand temperatures exceeded the pivotal temperature 52% (*z*‐test, *p* < 0.001) of the time (78% near the concrete wall versus 32% far away from the wall, *z*‐test, *p* < 0.001). The hatchery sand temperatures never reached the upper thermal tolerance of 36°C, while the sand temperatures on the beach exceeded the upper thermal tolerance 8% (*z*‐test, *p* < 0.001) of the time (Table 4). These sand temperatures do not consider the metabolic heat produced by embryos in nests. Assuming a 1.5°C increase in nest temperature due to metabolic heat, the percentage of hours above the thermal tolerance of nests on the beach would be 86% (*z*‐test, *p* < 0.001).

**TABLE 4:** Summary of the percentage of hours above the thermal tolerance (36.0°C) and pivotal temperature (30.3°C) of Olive Ridley Turtles and the mean sand temperatures (°C) with their respective ±SD at Hatchery El Banco and along the beach from November to December 2021.

Effect	Mean temperature (°C)	% hours > 36.0°C	% hours > 30.3°C
Sand temperature	35.2 ± 0.6	7.5	100.0
Hatchery sand temperature	30.4 ± 0.8	0.0	51.6
Concrete sand temperature	35.5 ± 0.5	13.5	100.0
Concrete hatchery sand temperature	30.8 ± 0.7	0.0	78.4
No concrete sand temperature	34.9 ± 0.5	1.6	100.0
No concrete hatchery sand temperature	30.0 ± 0.9	0.0	31.5

*Note:* Sand temperature is the temperature along the beach, while concrete sand temperature is the temperature in front of concrete structures and no concrete is the temperature in no close proximity of concrete structures or vegetation.

### 3.4 TSP and Sex Ratio

**FIGURE 7:**
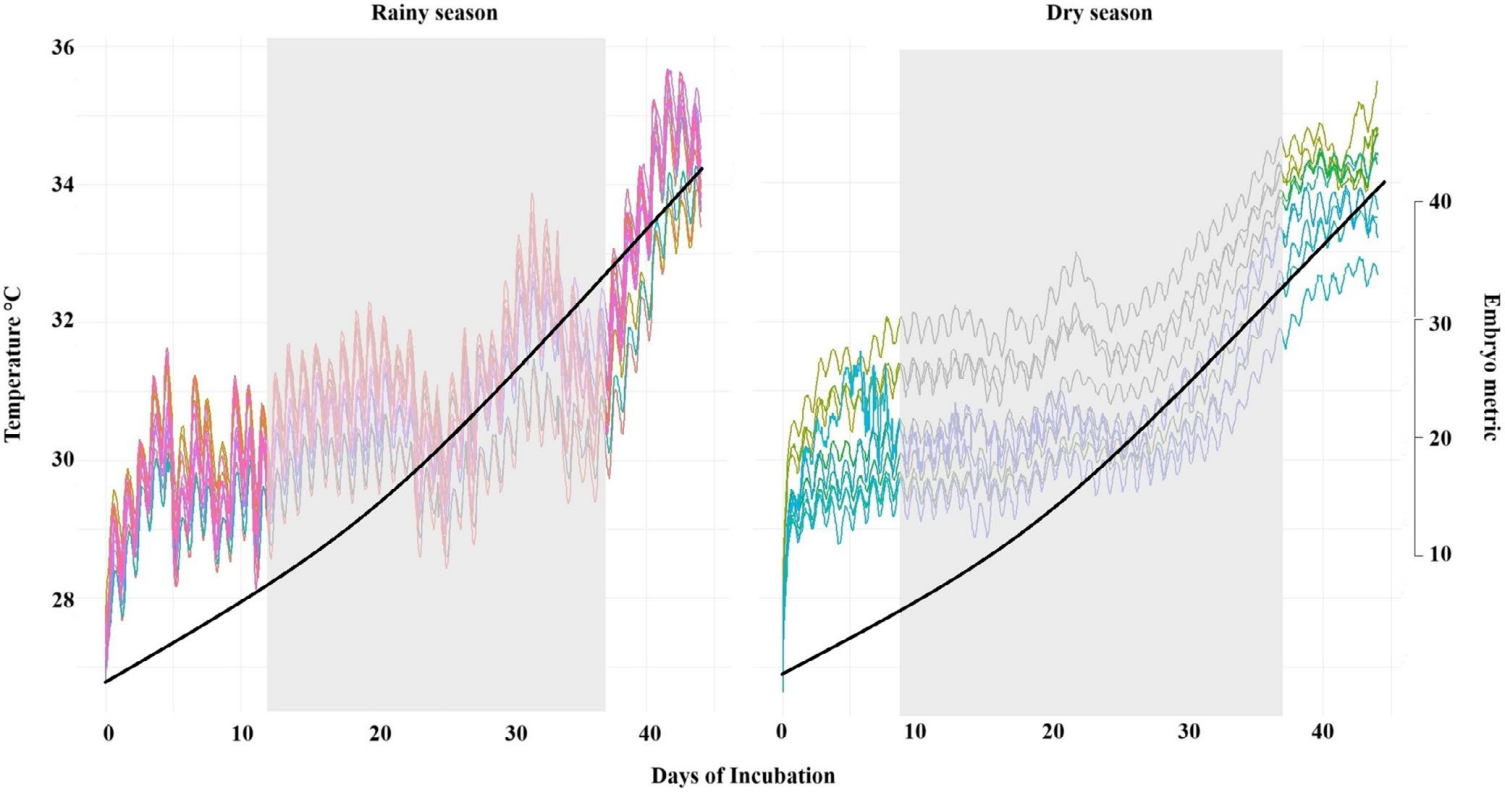
The hourly temperature of each nest separated by season. The gray area represents the mean TSP of the nests according to the development of the hatchlings. The black line represents the mean growth of the embryonic metric, as measured by the straight carapace length (mm).

The TSP for each nest varied from starting on day 9–15 and ending on day 33–37. The TSP was also slightly longer during the dry season (23.5 days ±0.5 SD, *n* = 10, *t*‐test, *p* < 0.001) compared to the rainy season (22 ± 0.4 days, *n* = 12, *t*‐test, *p* < 0.001). The hatchlings were estimated to be smaller during the dry season (mean straight carapace length of 40.6 mm ±2.3, *n* = 360, *t*‐test, *p* < 0.001) than in the rainy season (42.1 ± 2.4, *n* = 850, *t*‐test, *p* < 0.001) (Figure 7).

**FIGURE 8:**
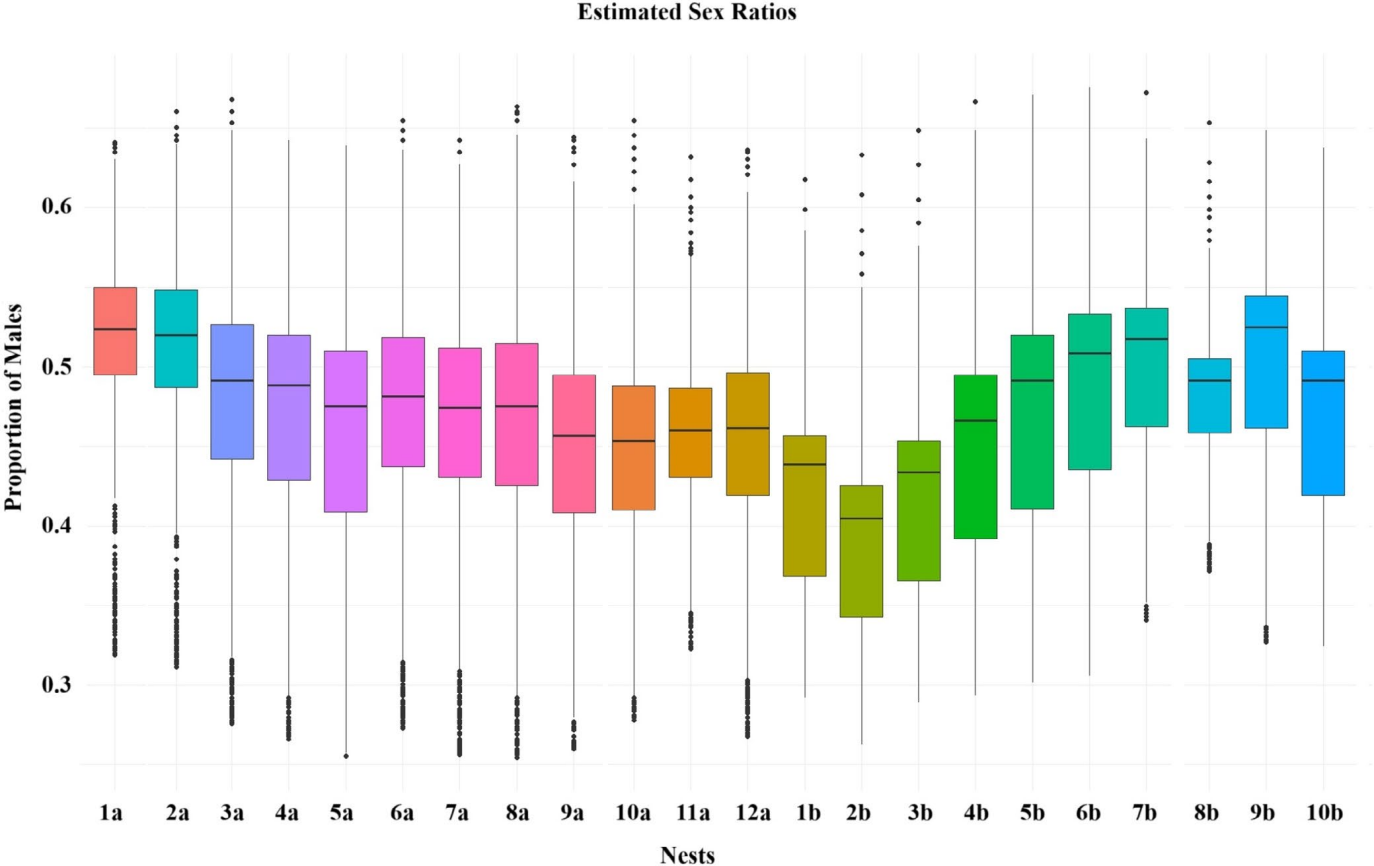
Estimated proportion of male hatchlings per nest. Box plots show the modeled sex ratio (proportion of males) for each nest based on nest‐specific thermal profiles during the TSP. Letters “a” and “b” represent nests from two distinct nesting seasons (rainy and dry, respectively). The horizontal line in each plot indicates the median proportion of males. The whiskers represent the data that falls out of the main group, and the black dots indicate the outliers. Colors differentiate nests visually and do not reflect temperature or location.

Almost all (90%, binomial test, *p* < 0.001) of the 40 dead hatchlings (sampled only during the rainy season) were females, and also based on the sex ratio estimations, fewer males (0.46 ± 0.03, *z*‐test, *p* < 0.001) and more females (0.54 ± 0.03, *z*‐test, *p* < 0.001) were expected to have hatched (Figure 8).

## 4 Discussion

The results of this study provide insights into the dynamics of nest temperature in a sea turtle hatchery. Nest temperatures in the hatchery frequently exceeded the pivotal temperature of Olive Ridley Turtles, but rarely the upper thermal tolerance limit. Despite our relatively modest sample size, it was clear that two factors produced higher nest temperatures: proximity to the concrete hatchery wall and a relatively large number of eggs in the nest. There was a much less pronounced effect of seasonal variation. Nevertheless, incubation temperatures decreased with increasing ambient air temperature and increased with rainfall, which contradicts the findings for in situ nests (Laloë et al. 2016; Pusapati et al. 2021; Flores and De La Cruz 2023).

Our research revealed that at the El Banco hatchery, nests remain above the pivotal temperature for a portion of the thermosensitive period during incubation. These elevated temperatures could suggest a female‐skewed sex ratio among hatchlings produced in the hatchery (Reneker and Kamel 2016; Casthelogue et al. 2018; Tello‐Sahagún et al. 2023). This finding is further supported by the analyses of the dead hatchlings, most (90%) of which were female. Contradicting this finding, our sex ratio estimations show a slight female bias but lower than found in other areas (Hernández‐Echeagaray et al. 2012; Dornfeld et al. 2015). This discrepancy between measured and modeled sex ratios has been documented in previous studies (Fuentes et al. 2017). It is likely due to the complex nature of sex determination, which can be influenced by microclimatic conditions and population‐specific pivotal temperatures, making accurate modeling more challenging (Girondot, Monsinjon, and Guillon 2018; Young et al. 2023; Girondot 2024). In contrast, the measured sex ratio (based on a subset of hatchlings) could be influenced by potential sex‐biased mortality (Kobayashi et al. 2017; Santidrián Tomillo 2022).

The maximum thermal tolerance of 36°C for embryos was not exceeded in the hatchery, and therefore, we assumed nests were not exposed to extreme temperatures over longer periods that would significantly threaten hatchling survival. However, other studies have shown that thermal limits vary among populations, which could mean that hatchery embryos may still be exposed to compromising temperatures (Howard et al. 2015; Laloë et al. 2017; Pusapati et al. 2021), but less extreme than in situ temperatures. Nevertheless, embryos also have mechanisms to buffer them against exposure to high temperature conditions (above thermal tolerance) for a shorter time (Tedeschi et al. 2016; Bentley et al. 2017). Systematic monitoring of nesting sites should be implemented to create better conservation programs (Türkozan et al. 2023).

### 4.1 Distance to Hatchery Wall

Our observations revealed that nests closer to the hatchery walls exhibited higher temperatures than those farther away. This temperature variation can be attributed to using concrete for the hatchery walls. Higher sand temperatures in the vicinity of concrete walls compared to sand temperatures in locations farther away from concrete walls have also been found on sea turtle nesting beaches (Ariano‐Sánchez et al. 2023). While the manual for the ex situ incubation of sea turtle eggs in El Salvador advises against using concrete as a building material for hatcheries (Ministerio de Medio Ambiente y Recursos Naturales 2008), there are still many hatcheries along the Pacific coast of Guatemala with concrete walls. However, different materials with lower heat capacity, such as wood, bamboo, or sandbags, are recommended to mitigate elevated nest temperatures (Ministerio de Medio Ambiente y Recursos Naturales 2008; Mohajerani et al. 2017). These alternatives are less durable than concrete, and since most sea turtle hatcheries in Guatemala are already built with concrete walls, a practical alternative to costly wall removal could be managing nest temperature by utilizing the distance from the wall as a tool.

### 4.2 Other Factors

Daily fluctuations in nest temperatures align with studies that indicate nest temperature naturally fluctuates throughout incubation, with shallow nests exhibiting daily variations in both amplitude and mean temperature (Booth 2018; Stokes et al. 2024). Further, we observed an increase in nest temperature throughout the incubation period, likely due to metabolic heat generated by embryo development (Deeming 2004; Sandoval et al. 2011; Gammon et al. 2020). The average temperature difference between the early and the late thirds of the incubation period was 3.6°C, higher than a review of the metabolic heat of sea turtle embryos, which found an average increase of 2.5°C (Gammon et al. 2020). As also shown in other studies, we found that the number of eggs per nest influenced nest temperature (Broderick et al. 2001; Chan et al. 2006), as more eggs generate more metabolic heat, raising nest temperatures in the middle and late thirds of incubation (Broderick et al. 2001). Splitting the nests in half (from 80 to 100 eggs to around 40–50 eggs) has been shown to reduce nest temperatures (Clarke et al. 2021) but may increase digging effort for hatchlings during emergence (Rusli and Booth 2016). However, some hatcheries manually dig out the hatchlings from the nest, but this practice may be a severe modification of an important part of hatchling behavior. Varying the number of eggs per nest could be implemented to modify nest temperatures in the hatchery. However, this also has disadvantages because fewer eggs per nest will reduce hatchery capacity.

We found that nest temperature was negatively correlated with air temperature and positively correlated with precipitation. Although opposite trends have been observed in in situ nests, it is often assumed that hatchery nests are somewhat shielded from external weather conditions (Laloë et al. 2016; Flores and De La Cruz 2023). Furthermore, the low thermal conductivity of sand and the shading provided by the hatchery may delay nest temperature responses to air temperature, potentially explaining the negative relationship (Tetteh et al. 2024). While precipitation events have been shown to reduce nest temperatures in in situ conditions (Laloë et al. 2016; Pusapati et al. 2021; Flores and De La Cruz 2023), the shade cloth in hatcheries may delay moisture infiltration into the sand, and since moist sand retains heat more effectively, this could cause a lag between rainfall and detectable cooling (Smits et al. 2010).

Precipitation affected nest temperature, yet we observed no temperature difference between the rainy and dry seasons. This may be due to random irrigation practices during the dry season to cool the sand, as noted by hatchery workers, though no official record exists. The hatchery also uses palm leaves to shade buildings as an extra shading resource during the dry season, as artificial shading is a proven method to reduce nest temperatures during warmer, drier times (Patino‐Martinez et al. 2012; Wiggins et al. 2023). Hatcheries can employ shading, irrigation, and other strategies to offset weather impacts, but monitoring nest temperature remains essential to address potential climate impacts effectively.

Other studies have reported temperature variation within sea turtle nests (Kaska et al. 2000; Luna et al. 2000; Booth and Astill 2001). In our study, we placed seven loggers at different positions within each nest, but were able to evaluate further just the top, center, and bottom positions. Although the position within the nest was not a significant factor in our statistical model, a visual comparison showed that the data logger placed in the middle of the nest recorded slightly higher temperatures during the TSP and the late third of development. This increase is likely due to the metabolic heat generated by developing embryos (Booth and Astill 2001; Gammon et al. 2020). During the early third, it was expected that the center of the nest would be cooler due to its insulation from external heat sources and the weak metabolic heat production at the beginning of development (Booth 1998; Gammon et al. 2020).

### 4.3 Comparison Ex Situ to In Situ

We found that sand temperatures in the hatchery were significantly lower than in situ sand temperatures, probably due to the hatchery being shaded and not being exposed to direct sun like in situ nests would be. The in situ sand temperatures were above the pivotal temperature the whole time, meaning that the in situ nests would produce only female hatchlings. However, since in situ sand temperature measurements do not reflect the actual temperatures in a sea turtle nest, we assume that metabolic heat would increase temperatures by 1.5°C in the nest (Gammon et al. 2020). Adopting these extrapolated temperatures, we estimated that the hatching success of nests incubated in situ at the nesting beach would be low. Another study has demonstrated that nests may hatch naturally in the area, but also in that study the hatching success was low and only based on the monitoring of four protected nests: one nest failed to hatch entirely, while the remaining three showed low to very low hatching rates (Morales‐Mérida et al. 2023). This strongly suggests that hatcheries are an important conservation tool to secure the survival of the sea turtle population on the Pacific coast of Guatemala and very likely also on other sea turtle nesting beaches in Latin America. For instance, in situ sex ratios in a study in Brazil showed an average female proportion of 0.9 (*n* = 55 nests) (Casthelogue et al. 2018). Ongoing changes in climate with higher temperatures are likely to accelerate the situation (Heppell et al. 2022; Hays et al. 2023). Additionally, the limited alternative income opportunities for low‐income coastal communities along the Pacific coast could also restrict any efforts to return the species to in situ nesting (CONAP 2018; MARN and PNUD 2018; Muccio 2019). Relying on ex situ methods to conserve a whole population implicates several limitations. The establishment, maintenance, and daily operation of hatcheries is costly and only possible if adequate funds are available and with highly committed operators around (Martins et al. 2021). The other severe limitation of ex situ conservation is the lack of genetic and behavioral adaptation to natural conditions (Mrosovsky 2006; Santidrián Tomillo et al. 2021; Robledo‐Avila et al. 2022). However, organisms must adapt to cope with ongoing climate change; otherwise, they may have to move away from their original habitat (Santidrián Tomillo et al. 2021). Other species have been seen to modify their behavior to adapt to changing environmental conditions. For instance, the three‐linked skinks and painted turtles search for shaded areas for in situ nesting to compensate for the high temperatures (Telemeco et al. 2009; Refsnider and Janzen 2012). On the other hand, recent research indicates that phenological shifts in sea turtles are unlikely to offset the effects of climate change (Fuentes et al. 2023). Since the 1970s, almost no sea turtles have hatched in situ on the Pacific coast of Guatemala (MARN and PNUD 2018), emphasizing the need for long‐term nest temperature monitoring in hatcheries to improve ex situ management (Hays et al. 2003). Thus, research efforts should focus on managing human manipulation of eggs and hatchlings for the best possible outcome for the population.

### 4.4 TSP and Sex Ratio

The TSP was longer during the dry season compared to the rainy season. Even though it has been shown that higher temperatures hasten the embryo development (Valverde et al. 2010), the slower development during the dry season in our study could be attributed to the additional shading and irrigation the hatchery employs during this time, disrupting continuous temperature rise (Hill et al. 2015; Reboul et al. 2021). Although the TSP was longer, the temperatures remained slightly higher during the dry season, which explains the smaller size of the hatchlings. Higher incubation temperatures often result in smaller hatchlings due to reduced yolk absorption (Booth and Evans 2011). Thus, while hatchling size reflects thermal conditions, TSP duration may be more sensitive to the interaction between temperature and microenvironmental conditions, such as shading, sand grain size, and moisture content (Staines et al. 2019; Stewart et al. 2019; Lamont et al. 2020).

The estimated sex ratio during the study is slightly female‐biased. The natural ideal sex ratio is unknown. However, natural populations in Mexico and Costa Rica have exhibited a female bias, with hatchlings ranging from approximately 55%–80% female (Hernández‐Echeagaray et al. 2012; Dornfeld et al. 2015). This means that the hatchery may be producing fewer females than in natural conditions, even though, according to the in situ temperatures recorded in this study, the in situ conditions could potentially produce only females. While a female‐skewed ratio is assumed to benefit the population since one male can mate with several females, there is a critical point where too few males can threaten population viability (Heppell et al. 2022; Fuentes et al. 2023; Hays et al. 2023). Extreme feminization, defined as a population with more than 85% females, can have long‐term effects on population stability (Heppell et al. 2022). However, having too many males is assumed to reduce the population with fewer nesting females (Wibbels et al. 1998; Hays et al. 2017; Santidrián Tomillo et al. 2023). As such, maintaining appropriate thermal conditions within the hatchery is important to avoid exceeding viable thresholds and to preserve natural sex ratio dynamics.

## 5 Conclusions

Our study investigated how various factors affect temperature variations in Olive Ridley Turtle ( *Lepidochelys olivacea* ) nests within a hatchery. We generally observed that the nest temperature regime in the hatchery was appropriate to produce viable hatchlings, yet nest temperatures in the hatchery exceed the pivotal temperature for Olive Ridley Turtles 6%–21% of the TSP. However, the upper thermal tolerance limit was usually not reached (in only 0.1% of the measurements). We found that nest temperatures in situ would exceed the thermosensitive threshold 100% of the measurements. Therefore, due to the widespread and intense egg collection activities in Guatemala, we conclude that hatchery conservation efforts are particularly important for sea turtle populations on the Pacific coast of Guatemala. In the hatchery, we found that nests closer to the concrete hatchery wall had temperatures up to 1°C higher compared to nests farther away from the concrete wall, and nests with 30–40 more eggs experienced an average temperature increase of 0.7°C. Based on our results, we recommend considering changing the construction material for the hatchery walls or placing the nests farther away from the walls. We also recommend using the nest positions in hatcheries and the number of eggs per nest as active management tools, in addition to, for example, irrigation and shading, to regulate nest temperatures and, consequently, sex ratios in sea turtle hatcheries. However, optimal sex ratios are not yet well understood. Also, relying solely on hatcheries could reduce the population's adaptability to environmental changes. However, as long as in situ nesting conditions are unfavorable on the Pacific coast of Guatemala, hatcheries as a conservation measure will continue to be necessary to secure the population's survival. Additionally, our study underscores the need for careful hatchery management and highlights the importance of protecting sea turtles from climate change.

## Author Contributions

**Jennifer A. Carbonell Ellgutter:** data curation (lead), formal analysis (lead), investigation (lead), methodology (equal), visualization (lead), writing – original draft (equal), writing – review and editing (lead). **Ingrid Maria Bik:** investigation (supporting), writing – original draft (equal). **Frank Rosell:** conceptualization (equal), methodology (equal), supervision (equal), writing – review and editing (equal). **Hans Renssen:** conceptualization (equal), methodology (equal), supervision (equal), writing – review and editing (equal). **Lucy A. Hawkes:** methodology (equal), supervision (equal), writing – review and editing (equal). **Stefanie Reinhardt:** conceptualization (lead), methodology (lead), supervision (lead), writing – review and editing (equal).

## Conflicts of Interest

The authors declare no conflicts of interest.

## Data Availability

Data is available through the following DOI: 10.23642/usn.28286642.
